# Genetic Characterization of *Staphylococcus aureus*, *Staphylococcus argenteus*, and Coagulase-Negative Staphylococci Colonizing Oral Cavity and Hand of Healthy Adults in Northern Japan

**DOI:** 10.3390/pathogens11080849

**Published:** 2022-07-28

**Authors:** Mina Hirose, Meiji Soe Aung, Yusuke Fujita, Taisei Kato, Yukito Hirose, Shoko Yahata, Atsushi Fukuda, Masato Saitoh, Noriko Urushibara, Nobumichi Kobayashi

**Affiliations:** 1 Division of Pediatric Dentistry, Department of Oral Growth and Development, School of Dentistry, Health Sciences University of Hokkaido, Ishikari-Tobetsu 061-0293, Japan; minaniwa@hoku-iryo-u.ac.jp (M.H.); fujita-y@hoku-iryo-u.ac.jp (Y.F.); katotaisei@hoku-iryo-u.ac.jp (T.K.); 8.yaha.24@gmail.com (S.Y.); atsushi@hoku-iryo-u.ac.jp (A.F.); msaitoh@hoku-iryo-u.ac.jp (M.S.); 2 Department of Hygiene, Sapporo Medical University School of Medicine, Sapporo 060-8556, Japan; noriko-u@sapmed.ac.jp (N.U.); nkobayas@sapmed.ac.jp (N.K.); 3 Division of Fixed Prosthodontics and Oral Implantology, Department of Oral Rehabilitation, School of Dentistry, Health Sciences University of Hokkaido, Ishikari-Tobetsu 061-0293, Japan; yukito@hoku-iryo-u.ac.jp

**Keywords:** MRSA, *Staphylococcus argenteus*, *Staphylococcus capitis*, coagulase-negative *Staphylococcus*, oral cavity, hand, SCC*mec*, PVL, ACME, NRCS-A

## Abstract

The spread of methicillin resistance and virulence among staphylococci in the community poses a public health concern. In this study, we investigated the prevalence of *Staphylococcus* species colonizing the oral cavity and hand (skin) of healthy university students and their phenotypic and genetic characteristics in northern Japan. Among a total of 332 subjects, 6 and 110 methicillin-resistant and susceptible *Staphylococcus aureus* (MRSA and MSSA, respectively) isolates were recovered from 105 subjects. MRSA isolates were genotyped as CC5, CC8, CC45, and CC59 with SCC*mec*-IIa or IV, among which an isolate of ST6562 (single-locus variant of ST8) harbored SCC*mec*-IVa, PVL genes and ACME-I, which are the same traits as the USA300 clone. ST1223 *S. argenteus* was isolated from the oral cavity and hand of a single student. Coagulase-negative *Staphylococcus* (CoNS) was recovered from 154 subjects (172 isolates), and classified into 17 species, with *S. capitis* being the most common (38%), followed by *S. warneri* (24%) and *S. epidermidis* (15%), including nine *mecA*-positive isolates. *S. capitis* was differentiated into seven clusters/subclusters, and genetic factors associated with the NRCS-A clone (*nsr*, *tarJ*, *ebh*) were detected in 10–21% of isolates. The colonization of the USA300-like MRSA variant and *S. capitis* with the traits of the NRCS-A clone in healthy individuals was noteworthy.

## 1. Introduction

*Staphylococcus* inhabits the skin and mucous membrane of humans and animals, forming normal bacterial flora. In terms of coagulase production, the genus *Staphylococcus* is largely classified into coagulase-positive and -negative staphylococcus (CoPS and CoNS, respectively), and some species with the coagulase-positive/variable trait [1]. CoPS is a major pathogenic group, including *Staphylococcus aureus* and minor species represented by *S. argenteus* [2]. *S. aureus* is the most common pathogen that causes various staphylococcal diseases such as superficial skin infections, pneumonia, bacteremia, food poisoning, and toxic shock syndrome [3]. *S. argenteus*, which is classified into *S. aureus* complex (SAC), has been recognized as an emerging pathogen in humans and animals, causing diseases similar to those of *S. aureus* [4]. Although CoNS represents a less virulent group of *Staphylococcus*, some CoNS species represented by *S. epidermidis*, *S. hominis*, *S. haemolyticus*, and *S. capitis* are described as common causes of opportunistic and nosocomial infections [3].

Methicillin-resistant *S. aureus* (MRSA) and CoNS (MR-CoNS) have been known as major pathogens that cause healthcare-associated infections [1,5]. Nevertheless, during the past two decades, community-acquired MRSA (CA-MRSA) has spread worldwide, also causing diseases in immunocompetent individuals [6]. MR *Staphylococcus* carries in its chromosome an SCC*mec* (Staphylococcal Cassette Chromosome *mec*) element containing *mecA* that encodes PBP2′ (PBP2a) (Penicillin-binding protein) with low affinity to oxacillin/cefoxitin. The SCC*mec* is a large genetic element and classified into at least 15 genetic types [5,7], among which type I through V are commonly found in hospital-acquired (HA)-MRSA or CA-MRSA. Among the CA-MRSA, the ST8-SCC*mec*-IVa clone that was designated USA300 has been the most commonly distributed in the US and is spreading worldwide [8,9]. This clone characteristically produces Panton-Valentine leukocidin (PVL), which is associated with severe symptoms of infections, and harbors arginine catabolic mobile element (ACME), which is located adjacent to SCC*mec* and contributes to the enhancement of its adaptability and the colonization of bacteria to the host [9]. ACME was revealed to be distributed in other MRSA clones [10] and CoNS, mainly in *S. epidermidis* and *S. capitis*, and classified into three genotypes (I-III) as well as their truncated forms (e.g., II’) [11,12,13].

The colonization of *S. aureus*/MRSA or CoNS is associated with an increased risk of bloodstream infections and surgical site infections [14,15]. The distribution and spread of MRSA and multidrug-resistant MR-CoNS among healthy individuals have been documented in many studies [16,17]. Accordingly, to evaluate the potential risk of infections in community and healthcare settings, it is of significance to reveal the colonization status of *Staphylococcus* on patients as well as healthy individuals. Although nasal carriage of *Staphylococcus* is often intermittent [18], colonization in the oral cavity is more persistent [19] and its rate is comparable to that in anterior nares [20,21]. Therefore, the oral cavity/oro-pharynx is considered to have a significant role as a reservoir of staphylococci for its cross-infection and dissemination to other body sites [19,22]. In our previous studies on healthy children [23] and dental patients/staff [24] in northern Japan, the proportion of MR (*mecA*-positive) isolates among *S. aureus* and CoNS from the oral cavity ranged from 3–6% and 13–21%, respectively. However, between these studies, an evident difference was observed for the clonal lineages of MRSA and the prevalence of MR isolates in CoNS species; MR isolates were the most common in *S. epidermidis* from children, in contrast to *S. saprophyticus*/*S. haemolyticus* from dental patients. Furthermore, CC8 (ST6562)-SCC*mec*-IVa MRSA, which is related to the USA300 clone, and two clones of *S. argenteus* were identified from dental patients, presenting the need for their further monitoring, in addition to healthy adult populations that had not yet been examined. The present study was conducted to analyze the oral *Staphylococcus* colonizing healthy adults in northern Japan, with regard to their species and clonal structure, genetic traits, and antimicrobial resistance.

*S. capitis* is one of the CoNS species constituting normal bacterial flora in humans [1]. However, a methicillin-resistant *S. capitis* clone designated NRCS-A, which was first reported in France, has been shown to cause bloodstream infections in neonates in the neonatal intensive care unit (NICU) with high morbidity [25,26]. NRCS-A is a multiresistant clone having type-V-related SCC*mec*, and harbors some virulence factors represented by *nsr* that encode a bacteriosin conferring nisin resistance [27,28,29]. This clone is considered to be distributed worldwide, mainly in Europe, the United States, and Australia, as the emerging cause of nosocomial sepsis in neonates [26,30,31]. Though the equipment in the NICU such as the incubator seem to be a potential mediator of the NRCS-A clone, a role of colonization in medical staff and healthy individuals for the spread of this clone remains to be determined [32]. Further, *S. capitis* was recently revealed as the major CoNS species that secretes 6-thioguanine (6-TG), a purine analogue that suppresses the growth of *S. aureus* [33]. Because *S. capitis* was the dominant CoNS species in the present study, the prevalence of the genetic factors that are associated with NRCS-A and 6-TG biosynthesis was investigated to clarify the spread of such virulent strains and also the antagonism with *S. aureus* in colonization.

## 2. Results

### 2.1. Prevalence of Staphylococcal Isolates from Study Subjects

This research was conducted as an observational, cross-sectional study at Health Sciences University of Hokkaido, Ishikari-Tobetsu, Hokkaido, Japan. Eligible subjects were recruited among students who attended annual medical check-up at the university during a period from April to May 2021. Students who participated in this study belonged to six faculties/schools (pharmaceutical science, dentistry, nursing and social services, psychological science, rehabilitation science, dental hygiene school) and graduate schools of the university. From subjects who agreed to participate in this study, oral-cavity samples (saliva) and skin-swab samples from the hand were collected.

A total of 332 students participated in this study. The age rage of the participants was 18-45 years, and the average was 20.5 years. One hundred and eighteen CoPS isolates (116 *S. aureus* and 2 *S. argenteus*) were recovered from 105 students (32% of all the subjects), while 172 CoNS isolates were recovered from 154 students (46%) (Table 1 and Table 2). Among the 118 CoPS, 101 isolates (86%) were derived from the oral cavity, with 26 isolates (14%) being obtained from both the oral cavity and the hand. The CoNS isolates were mostly recovered from the hand (165/172; 96%). Six MRSA isolates were obtained from the oral cavities of four subjects and the hands of two subjects, indicating the isolation rate of MRSA as 2% for all the subjects and 6% for the CoPS-positive subjects. More MSSA isolates were derived from the oral cavity (n = 84) than the hand (n = 26). *S. argenteus* was detected in one student (21-year-old, male; 0.3% of all subjects), from both the oral cavity and the hand.

### 2.2. Genotypes and Antimicrobial Resistance of CoPS

Six MRSA isolates were genotyped into *coa*-IIa, -IIIa, and -VIIb, and ST5, CC8 (ST8 and ST6562), ST45, and ST59 (Table 2). SCC*mec* type IV was the most common and found in four isolates with four STs, while the type IIa SCC*mec* was only detected in ST5 MRSA. ST6562 (allelic profile: 3-3-1-1-4-739-3) is a single-locus variant of ST8 (allelic profile: 3-3-1-1-4-4-3). PVL and type I ACME were only detected in the ST6562 MRSA isolate with SCC*mec*-IVa. Two *S. argenteus* isolates were *mecA*-negative, and both were classified into *coa*-XV and ST1223.

One hundred and ten MSSA isolates were assigned to 12 *coa*-types, with *coa*-Vb and VIIb being the most frequent, while *coa*-IIa, IIIa, VIIa, and Xa were also common (Table S1). The MSSA isolates were differentiated into 23 STs, among which ST188 (CC1) was the most common, followed by ST12, ST508 (CC45), ST8 and ST15. In this study, we identified four novel STs (ST6921, ST6922, ST6923, ST6924), among which ST6922 was grouped into CC5, and ST6923 was a single-locus variant of ST188.

Most MRSA showed resistance to AMP, ERY, and LVX, and inducible resistance to CLI, while they were susceptible to other antimicrobials including anti-MRSA drugs such as VAN (Table 2). MSSA was susceptible to most of the antimicrobials. Only low resistance rates (4–25%) were observed against AMP, ERY, CLI, GEN, and LVX, more commonly in ST8/ST30, ST30/ST508, ST30, ST508, and ST188/ST432, respectively. The *S. argenteus* isolates were susceptible to all of the antimicrobials examined.

### 2.3. Species, Genotypes and Antimicrobial Resistance of CoNS

A total of 172 CoNS isolates were classified into 17 species including *Mammaliicoccus sciuri* (Table 3). *S. capitis* was the most dominant, accounting for 38% of CoNS (66/172), followed by *S. warneri* (24%), *S. epidermidis* (15%), and *S. hominis* (8%), all of which were isolated from both the oral cavity and the hand. *mecA* was detected in six isolates (3.5% of CoNS) that were identified as *S. capitis* (n = 2), *S. epidermidis* (n = 5), and *S. hominis* (n = 2). Among the SCC*mec* types identified, IV was the most common and found only in *S. epidermidis*. ACME was detected in *S. capitis* and *S. epidermidis*, with incidence rates of 55% (36/66) and 58% (15/26), respectively. Among *S. capitis*, most ACME belonged to type II’ (30/36), while types I (n = 6) and II (n = 4) were commonly detected in *S. epidermidis*.

The CoNS isolates showed generally high susceptibility rates (86–100%) to all of the antimicrobials examined, although a resistance rate of only 38% was found against FOF, due to the higher frequency of FOF resistance in *S. capitis* and *S. warneri* (Table S2). Isolates resistant to AMP, ERY, GEN, and LVX were more commonly detected in *S. epidermidis* and *S. warneri*.

### 2.4. Genetic Characterization of S. capitis

**Figure 1:**
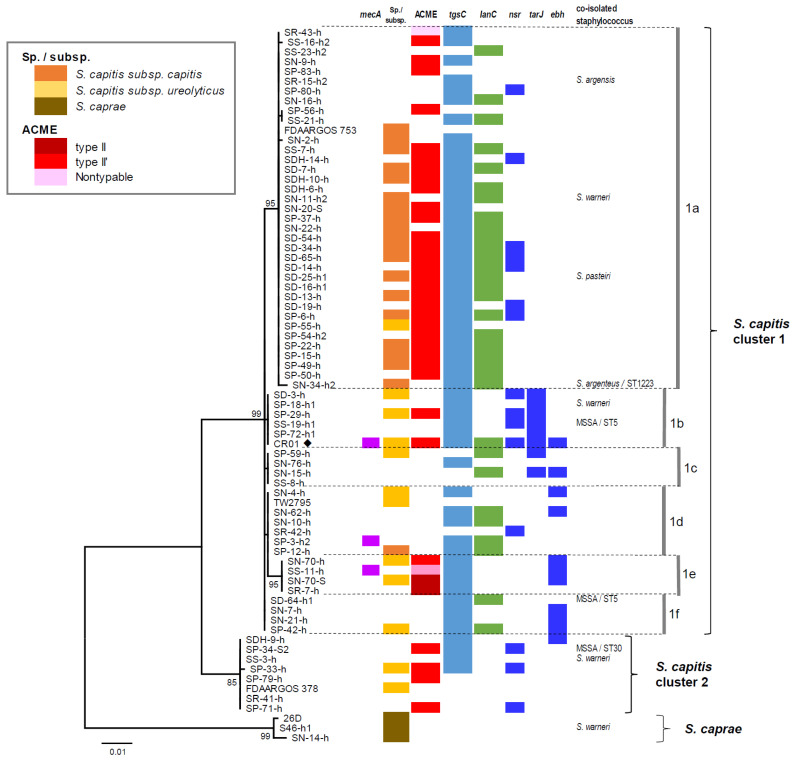
Phylogenetic dendrogram of partial *arcC* genes of *S. capitis* and *S. caprae* isolates linked with genetic characteristics. Dendrogram was constructed by maximum-likelihood method with MEGA.X program and statistically supported by bootstrapping with 1000 replicates, and genetic distances were calculated by Kimura two-parameter model. Variation scale is shown at the bottom. Percent bootstrap support is indicated by the values at each node (the values < 80 are omitted). Sequences of reference strains of *S. capitis* (FDAARAGOS 753, FDAARAGOS 378, CR01, TW2795) and *S. caprae* (26D) that were retrieved from GenBank database are added to the dendrogram. *S. capitis* clusters 1 and 2 with subclusters 1a–1d are shown on the right. *S. capitis* subspecies are shown only for isolates whose *hsp60* gene could be sequenced. Colors arranged in individual columns in the right side represent detection of *mecA*, ACME-*arcA*, *tgsC*, *lanC*, *nsr*, *tarJ*, *ebh* in the present study. For the reference strains, presence of these genes is shown for only CR01 marked with filled rhombus.

To understand the clonal diversity of *S. capitis*, which was the most frequently isolated among the CoNS species, we performed a phylogenetic analysis of *arcC*. As shown in Figure 1, 66 isolates were discriminated into two clusters 1 and 2, with six subclusters (1a–1f) in cluster 1. By the sequence analysis of the *hsp60* gene, 28 isolates were discriminated into *S. capitis* subsp. *capitis* (19 isolates) or *S. capitis* subsp. *ureolyticus* (9 isolates), though subspecies of other isolates were not identified due to the unsuccessful amplification of *hsp60* following the published PCR protocol [34]. *hsp60* sequences of *S. capitis* isolates exhibited >98.7% identity within individual subspecies, while 90.5–92.5% identity between these subspecies (data not shown). Although these subspecies were not evidently distinctive of clusters, subsp. *capitis* was mostly assigned to cluster1-subcluster 1a, and all other subclusters of cluster 1 and cluster 2 contained subsp. *ureolyticus*.

ACME was highly prevalent in the cluster-1 subclusters 1a and 1e, and cluster 2. *tgsC* was detected in 56 isolates (85% of *S. capitis* isolates) of all the cluster/subclusters. Among the five bacteriocin genes examined, only the gallidermine biosynthesis cluster gene (*lanC*) was detected in 31 isolates (47%), mostly in the cluster-1 subclusters 1a and 1d. Next, we attempted to detect the genetic factors associated with the NRCS-A clone, which has been known to have increased pathogenicity to neonates [25,26,27,28,29]. As a result, *nsr*, *tarJ* and *ebh* were identified in 14, 7, and 10 isolates (11–21%), respectively, and at least one of these genes was found in 27 *S. capitis* isolates (41%). The distribution of these genes was generally different depending on the genetic group; *nsr* in the cluster-1 subclusters 1a, 1b, and cluster 2; *tarJ* in the cluster-1 subcluster 1b; *ebh* in the cluster-1 subclusters 1c–1f. The coexistence of nsr and *tarJ* was observed in the cluster-1 subcluster 1b, which includes the NRCS-A prototype strain CR01. However, none of the isolates harbored all three genes. The nucleotide sequences of *tgsC*, *lanC*, *nsr*, *tarJ* and *ebh* that were determined for the representative isolates showed 97–100% identity to those of strain CR01 (data not shown), and were deposited to the GenBank database (Table S3).

### 2.5. Co-Isolation of S. aureus and CoNS

Among the 116 *S. aureus* isolates, 107 isolates (92%) were isolated solely from oral cavity or hand samples, without the isolation of CoNS. CoNS was co-isolated with nine *S. aureus* (1 MRSA, 8 MSSA) isolates and one *S. argenteus* isolate (Table 4). CoNS co-isolated with *S. aureus* belonged to five species, among which *S. capitis* and *S. warneri* were commonly found (three isolates each). Four *S. capitis* isolates that were co-isolated with MSSA and *S. argenteus* included those harboring *mecA* (n = 1), ACME (n = 2), and *tgsC* (n = 4). Among 66 *S. capitis* isolates, co-isolation with other *Staphylococcus* was found in nine isolates, which were all *tgsC*-positive and included four isolates with the gallidermine synthesis cluster gene.

## 3. Discussion

In the present study, we described the current status of staphylococcal colonization in the oral cavity and the skin of healthy adults in northern Japan, and revealed genetic characteristics of *S. aureus* and *S. capitis*. The prevalence of *S. aureus* (32%) and MRSA (2%) in our study, among university students of dentistry and other health science schools, was in line with those previously reported for healthy individuals; the isolation rate of *S. aureus* and MRSA from dental patients: oral cavity, 6–37% and 0–9%, respectively [35,36,37,38]; and dental students: any site of mouth/nose/skin, 15% and 3%, respectively [21]. Nevertheless, the present isolation rate of *S. aureus* was slightly lower than that in our latest research in the same study site, for dental patients and staff (44%) [24], suggesting a lower prevalence of *S. aureus* among healthy individuals unrelated to healthcare settings. Though the incidence of MRSA among *S. aureus* isolates in the present study (6 among 116 isolates; 5%) was comparable to that in our previous study (3 among 83 isolates; 3.6%), some genetic traits of MRSA were notable. First, two isolates belonged to SCC*mec*-II-ST5 (two isolates), while the remaining MRSA had SCC*mec*-IV (four isolates), which is most commonly carried by CA-MRSA [5,6]. ST5-MRSA with SCC*mec*-II has been predominantly known as HA-MRSA in Japan, eastern Asia and north America, designated the “New York/Japan clone” [39], and has been found to be a major MRSA strain in hospitals and the community in northern Japan [40,41]. In contrast, this strain has not yet been identified from the oral cavity of dental patients/staff or children in our study site [23,24], though it has been detected from community-associated infections [41]. The unexpected detection of colonizing ST5 SCC*mec*-II MRSA may imply the spread of the dominant HA-MRSA clone to healthy individuals in the community. Other STs or CC of MRSA isolates with SCC*mec*-IV, i.e., CC8, ST59, ST45, have been described as genotypes of common CA-MRSA clones [42], and detected in our previous studies for clinical isolates [41,43]. However, it was remarkable that ST6562 MRSA having SCC*mec*-IVa along with PVL genes and ACME-I was again identified, following its first detection in our previous study from the oral cavity of a 66-year-old dental patient [24]. This may suggest the potential dissemination of ST6562 (single-locus variant of ST8) MRSA among the community, despite a still low prevalence. Because the genetic traits of this clone are similar to those of the USA300 clone, which is predominantly CA-MRSA in the US [42], ST6562 is considered a variant of USA300 [24]. Though in Japan, the prevalence of the USA300 clone is still low, 5.1% of blood isolates of *S. aureus* were considered to be the USA300 clone in our previous study [43], and an increasing trend of this clone was also recently described by other researchers in Japan [44]. Thus, ST6562 is suggested to have emerged as a variant during the spread of USA300 in Japan. Still, the isolation of ST6562 MRSA from patients with infectious disease has not yet been reported. However, because the colonization of this clone may be a potential risk for severe disease due to PVL, as has been known for the USA300 clone, further surveillance may be necessary for colonizing and clinical isolates.

Among the MSSA isolated in the present study, the most commonly detected genotypes were ST5, ST8, ST12, ST15, ST30, ST188, ST508 (CC45), among which ST12, CC45, and ST188 were livestock-associated types [45,46]. Minor types found in this study, ST20, ST72, ST97, ST398, a part of CC5, and CC8 including ST72, were also related to animals [45]. Eventually, at least half of the MSSA isolates were related to animals, which was similarly observed in our previous study for dental patients/staff [24]. Therefore, a considerable part of MSSA colonizing healthy adults was suggested to be derived from animals in the present study, probably due to reduced pathogenicity to humans, while some isolates belonged to virulent clones represented by ST121 [47]. Furthermore, it was remarkable to identify ST1223 MS-*S. argenteus* from both the oral cavity and the hand of a single subject. The colonization of ST1223 *S. argenteus* was also described in the previous study in younger dental patients with 8–10-year-olds [24]. Despite a lower prevalence than *S. aureus*, *S. argenteus* has been identified among clinical isolates, as well as a cause of food poisoning in Japan [48,49]. Our present and previous findings provided evidence that the oral cavity and the skin could be reservoirs of *S. argenteus*.

The predominance of *S. capitis* among CoNS surpassing *S. epidermidis* was noteworthy, because the *S. epidermidis* has been the most prevalent CoNS species among those colonizing healthy individuals [17,23,50], as well as clinical isolates [12]. A similar trend of *S. capitis*, i.e., relatively higher frequency comparable to that of *S. epidermidis*, was observed in our previous study on colonizing *Staphylococcus* in dental patients/staff [24]. In addition, the incidence rate of ACME was 55%, which was comparable to that in *S. epidermidis* (58%) in the present study, and far higher than that in clinical isolates of *S. capitis* (7%) [12]. Although it is not clear whether this indicates an ecological change in the CoNS species among healthy individuals, it is possible that *S. capitis* with ACME might have spread as a colonizing strain due to the increased ability to persist on human skin. Furthermore, the production of bacteriocins and 6-TG [33,51] may have also contributed to the spread of *S. capitis*, overwhelming other CoNS species in the host. This may be supported in our present findings by the rather high incidence rates of *tgsC* (85%) and the gallidermin synthesis cluster gene (47%), and most of *S. capitis* isolates (62/66; 94%) were obtained without the co-isolation of *S. aureus*. However, the inhibiting effect on *S. aureus* in vivo conditions may not necessarily be explained by *S. capitis* 6-TG, because *S. aureus*/*S. argenteus* isolates from four specimens were co-isolated with *S. capitis* harboring *tgsC*.

While *S. capitis* has been known for its clinical importance as a cause of various infections including endocarditis, bacteremia, prosthetic joint infections, etc., the current crucial issue in public health is the emergence and spread of multidrug-resistant strains as well as the NRCS-A clone that causes neonatal sepsis [25,26,52]. As for traits specific to the NRCS-A clone, in silico prediction revealed the *nsr*, *ebh*, and *tarJ* genes, which were suggested to confer a competitive advantage to this clone in the neonatal gut [27,29]. In the present study, the *mecA*-positive (MR) rate in *S. capitis* was low (2/66; 3%). However, one or two of the *nsr*, *tarJ*, and *ebh* genes were detected in 10–21% of isolates. Though *nsr* was distributed to various genetic clusters/subclusters defined by *arcC*, the detection of *tarJ* and *ebh* was limited to some clusters. Particularly, isolates having both *nsr* and *tarJ* were classified into cluster-1 subcluster 1b, to which the prototype of NRCS-A (CR01 strain) was also assigned. Accordingly, it was revealed that *S. capitis* strains with genetically similar traits to the NRCS-A clone were colonizing in healthy adults. Although the reason for the emergence and worldwide endemicity of the NRCS-A clone is not evident, it is suggested that the distribution of some *S. capitis* lineages, such as cluster-1 subcluster 1b in the present study, may be related to the occurrence of the NRCS-A clone.

The present study revealed the colonization of USA300-like ST6525 MRSA and *S. argenteus*, and the presence of NRCS-A-clone-like *S. capitis* in healthy adults. Because these *Staphylococci* are considered to increase the risk of infections, further epidemiological surveillance of clinical and colonizing isolates may be necessary.

## 4. Materials and Methods

### 4.1. Study Subjects and Isolation of Staphylococcus

Saliva specimens of subjects were collected from the floor of the mouth by using a sterile cotton swab. A sterile cotton swab that was moistened with normal saline and rubbed on the palms and fingers was used as the hand swab specimen. All the swab samples were directly plated on CHROMagar Staph aureus (Kanto Chemical Industry Co., Ltd., Tokyo, Japan) and aerobically incubated at 37 °C for 48 h. *Staphylococcus*-like colonies were subcultured on blood agar plates followed by aerobic incubation at 37 °C overnight. For all the isolates grown on the plates, the partial 16S rRNA gene sequence was determined by Sanger sequencing with PCR products (approx. 1500-bp) as described previously [24]. The staphylococcal species of an isolate was identified as that showing >99% identity of the 16S rRNA sequence revealed by BLAST search (https://blast.ncbi.nlm.nih.gov/Blast.cgi, accessed on 31 January 2022). Individual isolates were stored in cryovials (Microbank, Pro-Lab Diagnostics, Richmond Hill, ON, Canada) at –80 °C and recovered when they were analyzed. DNA samples were extracted from cultured bacterial cells by the use of achromopeptidase (FUJIFILM Wako Pure Chemical Corp., Osaka, Japan). Briefly, 1–2 colonies from pure bacteria culture were dissolved in TNE 100µL in a 1.5 mL tube and were centrifuged at 10,000 rpm for 1 min. The supernatant was removed and 10 µL achromopetidase (10,000 U/mL) were added and mixed by vortex, then heated at 40 °C for 10 min in a water bath. Next, 50 µL each of 0.5 M KOH and 1M Tris–HCl (pH 6.8) were added and mixed by vortex. The 1.5 mL tube was centrifuged at 10,000 rpm for 1 min and the supernatant was used as a DNA template for the PCR reactions. The PCR mixture contained 200 µM dNTP, 0.5 µM of each primer, 1.25 U Ex Taq DNA polymerase (Takara Bio Inc., Shiga, Japan) and its buffer with Mg2+ (final conc. 2 mM), extracted bacterial DNA 1µL (approximately 2–3 ng), and sterile distilled water to a final volume of 25 µL. PCR was performed on a thermal cycler (Gene Atlas, ASTEC, Fukuoka, Japan) with the following conditions: preheating at 94 °C for 2 min, 30 cycles of denaturation at 94 °C for 30 s, annealing at 55 °C for 30 s and extension at 72 °C for 1 min, and a final extension at 72 °C for 5 min. PCR amplicons were analyzed for their product size using electrophoresis on a 1.5% agarose gel and the results were recorded by a gel documentation machine.

### 4.2. Antimicrobial Susceptibility Testing

For all the isolates, minimal inhibitory concentrations (MICs) within limited ranges were measured by the broth microdilution test using Dry Plate Eiken DP32 (Eiken, Tokyo, Japan) for 18 antimicrobials: oxacillin (OXA), ampicillin (AMP), cefazolin (CFZ), cefmetazole (CMZ), flomoxef (FMX), imipenem (IPM), gentamicin (GEN), arbekacin (ABK), erythromycin (ERY), clindamycin (CLI), vancomycin (VAN), teicoplanin (TEC), linezolid (LZD), minocycline (MIN), fosfomycin (FOF), levofloxacin (LVX), cefoxitin (FOX) and trimethoprim/sulfamethoxazole (SXT). Inducible clindamycin resistance (CLI-i) was determined by the D-zone test. Resistance was judged according to break points mentioned in the Clinical Laboratory Standards Institute (CLSI) standards [53] for most of the antimicrobials tested. For antimicrobial drugs whose breakpoints are not available in CLSI standards, we employed the European Committee on Antimicrobial Susceptibility Testing (EUCAST) breakpoint for FOF (32 mg/L, *Staphylococcus* spp.) [54], and a unique breakpoint for ABK (4 mg/L which is higher than the 2 mg/L, defined by the Japanese Society of Chemotherapy for respiratory infection), and a breakpoint of FMX (16 mg/L) defined by the Japanese Society of Chemotherapy for urinary tract infection [55].

### 4.3. Initial Genetic Characterization of Staphylococcal Isolates

For all the isolates, the presence of *nuc*, *mecA*, PVL genes, and ACME-associated *arcA* was confirmed by multiplex PCR assay as described by Zhang et al. [56]. In addition, PCR targeting the nonribosomal peptide synthetase (NRPS) gene was performed as previously described [57], to discriminate non-SAC species (*S. argenteus*, *S. schweitzeri*) from *S. aureus*. For all the methicillin resistant (*mecA*-positive) isolates, SCC*mec* type and subtype of SCC*mec*-IV were determined by multiplex PCR using previously published primers and conditions [58,59]. For all the ACME *arcA*-positive isolates, ACME type I, II, III, I’, and II’ was assigned by long-range PCR (LR-PCR) as described previously [12].

### 4.4. Genetic Typing and Analysis of S. aureus, S. argenteus, and S. capitis

The genotype based on the staphylocoagulase gene (*coa* type) of *S. aureus* and *S. argenteus* was determined by sequencing of partial *coa* (D1, D2, and the central regions), via PCR amplification with *coa*-ant1 and *coa*7 primers [60], and the subsequent search for *coa* type representing a highly similar *coa* sequence by BLAST. The sequence type (ST) of *S. aureus* and *S. argenteus* was determined according to the scheme of multilocus sequencing typing (MLST) [61].

For the genetic discrimination of two subspecies of *S. capitis*, i.e., subsp. *capitis* and subsp. *ureolyticus*, the partial *hsp60* gene sequence was determined by direct sequencing of the PCR product with primers described by Kwok et al. [34]. The subspecies was assigned based on high sequence identity (>98%) of the *hsp60* gene to that of prototype strains of *S. capitis* subsp. *capitis* (ATCC27840) or *S. capitis* subsp. *ureolyticus* (ATCC49324) [62]. Because the MLST scheme is not available for *S. capitis*, clonal diversity of *S. capitis* was analyzed by *arcC*, a housekeeping gene encoding carbamate kinase, which is included as one of the loci of the MLST scheme of most staphylococcal species. Partial *arcC* gene (approx. 500 bp) was amplified by PCR with primers designed in this study (Table S4), and its nucleotide sequence was determined by Sanger sequencing using BigDye Terminator v3.1 Cycle Sequencing kit (Applied Biosystems, Foster City, CA, USA) on an automated DNA sequencer (ABI PRISM 3100, Applied Biosystems, Foster City, CA, USA). A phylogenetic dendrogram of *arcC* was constructed by the maximum-likelihood method using the MEGA.X software, together with *arcC* sequence data of *S. capitis* reference strains retrieved from GenBank database. For *S. capitis* isolates, the presence of the following genes was detected by PCR with primers listed in Table S4: bacteriocin genes encoding Nisin J, epidermicin, gallidermine biosynthesis cluster, PSM beta peptidase, and capidermicin; NRCS-A clone-related genes *nsr*, *tarJ*, and *ebh* involved in nisin resistance, teichoic acid biosynthesis, and cell-wall-associated fibronectin binding, respectively. The *tgsC* gene, which is among the 6-TG biosynthetic genes and located in the middle of the gene cluster [33], was detected by PCR as described previously [63]. The nucleotide sequences of the above-mentioned genes were determined by PCR and direct sequencing.

## Figures and Tables

**Table 1:** Isolation of *Staphylococcus* from participants (n = 332) in this study.

Staphylococcal Species, Site of Isolation	No. of Subjects	No. of Isolates
*S. aureus*/*S. argenteus*		
oral cavity only	75	75
hand (skin) only	17	17
both oral cavity and hand	13	26
total	105	118
CoNS		
oral cavity only	7	7
hand (skin) only	140	151
both oral cavity and hand	7	14
total	154	172

**Table 2:** Genotypes and antimicrobial resistance profiles of MRSA/MSSA/*S.* *argenteus* isolates.

MRSA/MSSA/*S. argenteus*	Number of Isolates (Oral Cavity/Hand)	Genotype			PVL Gene/ACME Type	Number of Isolates Showing Resistance to Antimicrobials (%)								
		Coagulase Genotype	ST (CC/Variant)	SCC*mec* Type		OXA	FOX	AMP	CFZ	ERY	CLI-i	CLI-c	GEN	LVX
MRSA	2 (1/1)	*coa*-IIa	ST5 (CC5)	SCC*mec* IIa	-	2	2	2	1	2	0	2	0	2
	1 (1/0)	*coa*-IIIa	ST8 (CC8)	SCC*mec* IVh	-	1	1	1	1	1	0	1	0	1
	1 (0/1)	*coa*-IIIa	ST6562 (CC8)	SCC*mec* IVa	PVL(+)/ACME-I	1	1	1	0	1	0	0	1	1
	1 (1/0)	*coa*-VIIb	ST59 (CC59)	SCC*mec* IVg	-	1	1	1	1	1	0	1	1	0
	1 (1/0)	*coa*-VIIb	ST45 (CC45)	SCC*mec* IVa	-	1	1	1	0	0	0	0	0	1
total	6 (4/2)					6	6	6	3	5	0	4	2	5
MSSA	3 (2/1)	*coa*-Ia	ST672		-	0	0	0	0	0	0	0	0	0
	8 (5/3)	*coa*-IIa	ST5 (CC5)		-	0	0	1	0	1	1	0	0	0
	1 (1/0)	*coa*-IIa	ST863 (CC5)		-	0	0	0	0	0	0	0	0	0
	2 (1/1)	*coa*-IIa	ST6922 * (CC5)		-	0	0	0	0	0	0	0	0	0
	9 (8/1)	*coa*-IIIa	ST8 (CC8)		-	0	0	5	0	0	0	0	1	0
	1 (1/0)	*coa*-IIIa	ST6921 * (ST78 SLV)		-	0	0	0	0	1	0	0	0	0
	8 (5/3)	*coa*-IVa	ST30 (CC30)		-	0	0	8	0	5	5	0	0	0
	2 (2/0)	*coa*-Va	ST121 (CC121)		-	0	0	0	0	0	0	2	0	0
	2 (2/0)	*coa*-Vb	ST72 (CC8)		-	0	0	2	0	1	1	0	0	0
	13 (10/3)	*coa*-Vb	ST188 (CC1)		-	0	0	1	0	0	0	0	0	3
	2 (1/1)	*coa*-Vb	ST432		-	0	0	0	0	1	1	0	1	2
	1 (1/0)	*coa*-Vb	ST6923 * (ST188 SLV)		-	0	0	0	0	0	0	0	0	0
	7 (7/0)	*coa*-VIa	ST96		-	0	0	0	0	0	0	0	0	0
	6 (4/2)	*coa*-VIc	ST97		-	0	0	0	0	2	2	2	0	0
	12 (10/2)	*coa*-VIIa	ST12		-	0	0	0	0	0	0	0	0	0
	1 (0/1)	*coa*-VIIb	ST59		-	0	0	0	0	0	0	0	0	0
	5 (3/2)	*coa*-VIIb	ST45 (CC45)		-	0	0	2	0	1	1	0	1	0
	10 (8/2)	*coa*-VIIb	ST508 (CC45)		-	0	0	2	0	4	0	0	4	0
	3 (1/2)	*coa*-VIIb	ST398		-	0	0	0	0	2	2	0	0	0
	3 (3/0)	*coa*-VIIIa	ST20		-	0	0	1	0	0	0	0	0	0
	1 (1/0)	*coa*-VIIIa	ST6924 * (ST20 DLV)		-	0	0	1	0	0	0	0	0	0
	9 (7/2)	*coa*-Xa	ST15 (CC15)		-	0	0	4	0	0	0	0	0	0
	1 (1/0)	*coa*-Xa	ST2404 (ST15 SLV) (CC15)		-	0	0	0	0	0	0	0	1	0
total	110 (84/26)				-	0	0	27	0	18	13	4	8	5
***S. argenteus***	2 (1/1)	*coa*-XV	ST1223			0	0	0	0	0	0	0	0	0

None of the isolates showed resistance to ABK, CMZ, FMX, IPM, LZD, MIN, FOF, SXT, TEC and VAN. Abbreviations: ABK, Arbekacin; AMP, Ampicillin; CFZ, Cefazolin; CLI, Clindamycin; CMZ, Cefmetazole; ERY, Erythromycin; FMX, Flomoxef: FOF, Fosfomycin; FOX, Cefoxitin; GEN, Gentamycin; IPM, Imipenem; LVX, Levofloxacin; LZD, Linezolid; MIN, Minocycline; OXA, Oxacillin; SXT, Sulfamethoxazole-Trimethoprim; TEC, Teicoplanin; VAN, Vancomycin. * novel ST identified in this study.

**Table 3:** Prevalence of *mecA* and ACME in individual CoNS species.

CoNS Species	Number of Isolates *^1^			SCC*mec* Type of mecA-Positive Isolates (No. of Isolates) *^2^	ACME Type (No. of Isolates) *^2^
	Total (Oral Cavity/Hand)	*mecA* (+)	ACME (+)		
*S. capitis*	66 (3/63)	2	36	SCC*mec* III (1), SCC*mec* NT (1)	II’ (30), II (3), NT (3)
*S. warneri*	41 (4/37)	0	0		
*S. epidermidis*	26 (4/22)	5	15	SCC*mec* IV (4), SCC*mec* NT (1)	I (6), I’ (1), II (4), II’ (1), III (1), NT (2)
*S. hominis*	14 (1/13)	2	0	SCC*mec* NT (2)	
*S. pasteuri*	6 (0/6)	0	0		
*S. saprophyticus*	4 (1/3)	0	0		
*S. lugdunensis*	3 (0/3)	0	0		
*S. caprae*	2 (0/2)	0	0		
*S. haemolyticus*	2 (1/1)	0	0		
*S. argensis*	1 (0/1)	0	0		
*S. cohnii*	1 (0/1)	0	0		
*S. condimenti*	1 (0/1)	0	0		
*S. petrasi*	1 (0/1)	0	0		
*S. schleiferi*	1 (0/1)	0	0		
*S. succinus*	1 (0/1)	0	0		
*S. xylosus*	1 (0/1)	0	0		
*M. sciuri* *^3^	1 (0/1)	0	0		
CoNS total	172 (14/158)	9	51	SCC*mec* III (1), SCC*mec* IV (4), SCC*mec* NT (4)	I (6), I’ (1), II (7), II’ (31), III (1), NT (5)

*^1^ Both SCC*mec* and ACME and were detected in one *S. capitis* and three *S. epidermidis* isolates. *^2^ NT, non-typable. *^3^ This strain has intrinsic *mecA*-homologue and showed resistance to oxacillin.

**Table 4:** Co-isolation of *S. aureus*/*S. argenteus* and CoNS.

Site of Isolation	*S. aureus*/*S. argenteus*-ST (No.)	CoNS Species (No.) Co-Isolated	Genetic Traits of CoNS
oral cavity	MRSA-ST59 (1)	*S. warneri* (1)	*mecA*-, ACME-, *tgsC*-, *lanC*-
oral cavity	MSSA-ST30 (1)	*S. capitis* (1)	*mecA*-, ACME+, *tgsC*+, *lanC*-
hand	MSSA-ST508 (2)	*S. warneri* (2)	*mecA*-, ACME-, *tgsC*-, *lanC*-
hand	MSSA-ST5 (1)	*S. capitis* (1)	*mecA*+, ACME-, *tgsC*+, *lanC*+
hand	MSSA-ST5 (1)	*S. capitis* (1)	*mecA*-, ACME+, *tgsC*+, *lanC*-
hand	MSSA-ST672 (1)	*S. epidermidis* (1)	*mecA-*, ACME+, *tgsC*-, *lanC*-
hand	MSSA-ST8 (1)	*S. pasteuri* (1)	*mecA*-, ACME-, *tgsC*-, *lanC*-
hand	MSSA-ST398 (1)	*S. lugdunensis* (1)	*mecA*-, ACME-, *tgsC*-, *lanC*-
hand	*S. argenteus*, ST1223 (1)	*S. capitis* (1)	*mecA*-, ACME-, *tgsC*+, *lanC*+
oral cavity	MRSA (3), MSSA (83)	None	
oral cavity	*S. argenteus*, ST1223 (1)	None	
hand	MRSA (2), MSSA (19)	None	

## Data Availability

Not applicable.

## Supplementary Materials

The following are available online at https://www.mdpi.com/article/10.3390/pathogens11080849/s1, Table S1: Prevalence of coagulase genotypes among MSSA, MRSA and *S. argenteus* isolates; Table S2: Antimicrobial resistance profile of CoNS isolates detected in this study (n = 172); Table S3: Nucleotide sequences of *S. capitis* isolates determined in the present study and GenBank accession numbers; Table S4: Primers used for the analysis of *S. capitis* in this study.
