# Supplementary material for: Genetic Characterization of Staphylococcus aureus, Staphylococcus argenteus, and Coagulase-Negative Staphylococci Colonizing Oral Cavity and Hand of Healthy Adults in Northern Japan
Source: Pathogens. 2022 Jul 28;11(8):849. doi: 10.3390/pathogens11080849 (PMC9413425; doi:10.3390/pathogens11080849)
Supplement: Supplementary file 1 [file pathogens-11-00849-s001.zip › pathogens-1831805-supplementary.pdf]

Table S1. Prevalence of coagulase genotypes among MSSA, MRSA and *S. argenteus* isolates

Coagulase genotype	MSSA	MRSA	*S. argenteus*	Total	%
Ia	3	0	0	3	2.5
IIa	11	2	0	13	11
IIIa	10	2*	0	12	10.2
IVa	8	0	0	8	6.8
Va	2	0	0	2	1.7
Vb	18	0	0	18	15.3
VIa	7	0	0	7	5.9
VIc	6	0	0	6	5.1
VIIa	12	0	0	12	10.2
VIIb	19	2	0	21	17.8
VIIIa	4	0	0	4	3.4
Xa	10	0	0	10	8.5
XV	0	0	2	2	1.7
	110	6	2	118	

*one isolate is USA300-like clone.

Table S2. Antimicrobial resistance profile of CoNS isolates detected in this study (n=172)

staphylococci species (17)	Total (n=172)	Number of isolates showing resistance to antimicrobials (%)														SCC*mec* type of *mecA*-positive isolates
		OXA	FOX	AMP	CFZ	CMZ	FMX	ERY	CLI-i	CLI-c	GEN	MIN	FOF	SXT	LVX	
S. capitis subsp. *capitis*	66	2	2	4	1	1	1	3	0	0	4	0	37	0	1	SCC*mec*III (1), SCC*mec*-NT(classC2, *ccr*C, *ccr*A1B1) (1)
S. warneri	41	0	0	6	0	0	0	8	2	6	5	0	15	0	2	
S. epidermidis	26	5	5	5	0	0	0	6	2	4	6	0	4	0	5	SCC*mec*IV (4), SCC*mec*NT(classA, classB, *ccr*A2B2) (1)
S.hominis	14	2	2	3	0	0	0	2	2	0	0	0	1	2	2	SCC*mec*NT (classA) (2)
S. pasteuri	6	0	0	1	0	0	0	1	1	0	2	0	2	0	0	
S. saprophyticus	4	0	0	0	0	0	0	2	0	2	0	0	2	0	0	
S. lugdunensis	3	0	0	1	0	0	0	0	0	0	1	0	0	0	0	
S. caprae	2	0	0	1	0	0	0	0	0	0	1	0	2	0	1	
S. haemolyticus	2	0	0	0	0	0	0	0	0	0	0	0	0	0	0	
S. argensis	1	0	0	0	0	0	0	0	0	0	0	0	0	0	0	
S. cohnii	1	0	0	0	0	0	0	0	0	0	0	0	0	0	0	
S. condimenti	1	0	0	0	0	0	0	0	0	0	0	0	0	0	0	
S. petrasi	1	0	0	0	0	0	0	0	0	0	0	0	0	0	0	
S. schleiferi	1	0	0	0	0	0	0	0	0	0	0	0	0	0	0	
S. succinus	1	0	0	1	0	0	0	0	0	0	0	0	1	0	0	
S. xylosus	1	0	0	1	0	0	0	1	0	1	0	0	1	0	0	
M. sciuri	1	1	0	0	0	0	0	0	0	0	0	0	1	0	0	
TOTAL	172	10 (5.8)	9 (5.2)	23 (13.4)	1 (0.6)	1 (0.6)	1 (0.6)	23 (13.4)	7 (4.1)	13 (7.6)	19 (11.0)	0 (0)	66 (38.4)	2 (1.2)	11 (6.4)	SCC*mec*III (1), SCC*mec*IV (4), SCC*mec*NT (4)

None of the isolates showed resistance to ABK, IPM, LZD, SXT, TEC and VAN.

Abbreviations: ABK, Arbekacin; AMP, Ampicillin; CFZ, Cefazolin; CLI, Clindamycin; CMZ, Cefmetazole; ERY, Erythromycin; FMX, Flomoxef: FOF, Fosfomycin; FOX, Cefoxitin; GEN, Gentamycin; IPM, Imipenem; LVX, Levofloxacin; LZD, Linezolid; MIN, Minocycline; OXA, Oxacillin; SXT, Sulfamethoxazole-Trimethoprim; TEC, Teicoplanin; VAN, Vancomycin.

Table S3. Nucleotide sequences of *S. capitis* isolates determined in the present study and GenBank accession numbers.

Gene	Isolate ID (no. of isolates)	GenBank accession no. (Isolate ID) (no. of isolates)
tgsC	SP-18-h1, SP-29-h, SN-76-h, SN-70-S, SS-3-h, SD-16-h1, SD-14-h, SP-6-h, SP-22-h, SN-2-h, SD-25-h1, SD-3-h, SP-15-h, SN-20-S, SN-11-h2, SN-16-h (n=16)	ON684330-ON684336 (SP-15-h, SD-25-h1, SN-16-h, SP-6-h, SN-76-h, SP-29-h, SS-3-h) (n=7)
ebh	SS-11-h, SDH-9-h, SN-15-h (n=3)	ON684337-ON684339 (SS-11-h, SDH-9-h, SN-15-h) (n=3)
lanC	SD-64-h1, SN-62-h, SN-10-h (n=3)	ON684340-ON684342 (SD-64-h1, SN-62-h, SN-10-h) (n=3)
tarJ	SD-3-h, SN-15-h, SP-72-h, SS-19-h1, SP-18-h1, SP-29-h, SP-59-h (n=7)	ON684343-ON684347 (SN-15-h, SP-18-h, SP-72-h1, SD-3-h, SP-29-h) (n=5)
nsr	SP-33-h, SP-34-S2, SP-71-h, SD-19-h, SS-19-h, SD-3-h, SP-29-h, SD-34-h, SD-14-h, SP-6-h, SDH-14-h, SP-80-h, SD-65-h (n=13)	ON684348-ON684353 (SP-80-h, SD-34-h, SD-65-h, SP-6-h, SP-29-h, SD-3-h) (n=6)

Table S4. Primers used for the analysis of *S. capitis* in this study

Target gene	primer	Nucleotide sequence	Product size	Name of target gene	purpose	Reference
tgsC	tgsC-1	(+) GATAAATTAGACATATGTGA	560 bp	6-TG biosynthesis cluster	PCR detection, sequencing	Osada et al., 2022 [45]
	tgsC-2	(-) CTATATTAGAATTAAAACCAAATG				
S.capi-arcC	Scapi-arcC1	(+) CCAACTAAACCGATTGGTCC	528 bp	*S.capitis* carbamate kinase	PCR detection, sequencing	this study
	Scapi-arcC2	(-) TGCTTGCTTGTCGAACGCTT				
	Scapi-arcC1a	(+) CAATTATTACTCGTGTAGA	542 bp		ditto	
	Scapi-arcC2a	(-) TTATTTGTGAATATGTGTACC				
	Scapi-arcC1b	(+) GAAGTTGATAAAGATGATCC	550 bp		ditto	
	Scapi-arcC2b	(-) GTGTACCTGCTTGCTTGTCG				
S.cap-nsj	S.cap-nsj1	(+) TCAGAATTCACTACAATGGT	356 bp	Nicin J	PCR detection	this study
	S.cap-nsj2	(-) TTAAGATTAGCAGCCTGAAC				
S.cap-epd	S.cap-epd1	(+) AGTGTAATGAATACGTATTC	296 bp	epidermicin	ditto	
	S.cap-epd2	(-) GAGCTAGAAACTACTAAAGC				
S.cap-gald	S.cap-gald1	(+) CTTTTAATAGGAGTTGATG	534 bp	gallidermin biosynthesis cluster	ditto	
	S.cap-gald2	(-) CACATTACAAATAAACCTAG				
	SC-galid-lanC-F1	(+) ATCGAGATAAAGAGAAATTTGTAC	1400 bp		sequencing	
	SC-galid-lanC-R1	(-) CTTAAATGTTTACTATTGGTTAACGC				
S.cap-psmb1	S.cap-psmb1	(+) TAGAAACACAGTCGATGCTG	980 bp	PSM beta petidases	PCR detection	
	S.cap-psmb2	(-) CTAATTTTGCCCAGTCATGG				
S.cap-capid	S.cap-capid1	(+) CATACAAAATCACTACCCAT	354 bp	capidermicin	ditto	
	S.cap-capid2	(-) GTTGAATGTTTCACACAATATG				

Table S4. continued

*nsr**	S.cap-nsr-F	(+) GGAGATATGGGACCTATGATTGCA	224 bp	nisin resistance protein	PCR detection	Simoes et al., 2016
	S.cap-nsr-R	(-) GCTGTAAkTTCwCCkGAACTkGC				[27]
	SC-nsr-F1	(+) GTGATGTTGTCTTTTGGGATC	1104 bp		sequencing	This study
	SC-nsr-R1	(-) ACGTCTAGGCAAAGCAATCATGC				
	SC-nsr-F2	(+) GAAAGGATTAAATTACTAATG				
	SC-nsr-R2	(-) GACGATGTAATCTCTTATCTAGC				
	SC-nsr-R3	(-) AGCTACTCTTAGAGCTTCCT				
*ebh**	S.cap-ebh-F	(+) GCCATTGGAACATTTTCGAC	491 bp	cell wall associated fibronectin binding protein	PCR detection	This study
	S.cap-ebh-R	(-) TCTAAAGGCGGTTGAACAAG				
	SC-ebh-Beg1	(+) CATTATGGTCAATTTTAAGCACACGA	900 bp		sequencing	
	SC-ebh-sR1	(-) TTAACGCTTCTAGTAGAACGG				
	SC-ebh-IF1	(+) CTTGGTAATAGTGAAAATTC	1200 bp			
	SC-ebh-IR1	(-) CATAACTTAAAGGATCGTCGGC				
	SC-ebh-IF2	(+) AGCTTGATTTGATGTACCAAC				
	SC-ebh-IR2	(-) CATCGGTTCAACATATAATTGG				
*tar-J**	S.cap-tarJ-F	(+) TCAATTGGTAGCACCAAGAC	281 bp	teichoic acid biosynthesis	PCR detection	This study
	S.cap-tarJ-R	(-) GCTATAACTTCATCTTGTTCC				
	SC-tarJ-F1	(+) CGACCTTAAAGTAGCAAACTC	1097 bp		Sequencing	
	SC-tarJ-R1	(-) TCTTTATTGTTTGCTGTCTGG				
	SC-tarJ-F2	(+) GTCTTTATGGAACATGATCG				
	SC-tarJ-F3	(+) ATTTGAATGCGTAGGTGGGA				

* NRCS-A clone specific genes
